# Design, analysis and reporting of active-control randomised trials: a systematic review

**DOI:** 10.1186/1745-6215-14-S1-O123

**Published:** 2013-11-29

**Authors:** Lang'o Odondi, Chris Metcalfe, Jonathan Sterne

**Affiliations:** 1 University of Bristol, Bristol, UK

## Background

Active-control randomised trials are typically conducted to evaluate the benefit of a novel intervention relative to an established treatment. The appropriate design, analysis and conclusions depend upon whether the novel intervention is anticipated to be superior or non-inferior on the primary outcome measure. But despite their widespread application, active-control studies are often poorly understood, improperly applied, and incorrectly interpreted.

## Methods

We systematically review recently published active-control trials in a high-impact journal, to appraise their design, analysis and the conclusions drawn. All trials published in 2010 were identified and data extracted in duplicate using a standard proforma. Meta-analyses examined differences in the average treatment effect between superiority and non-inferiority studies.

## Results

Thirty-seven studies met our inclusion criteria; 12 designed as non-inferiority studies. Non-inferiority studies did not have larger sample sizes (median=702) compared to superiority studies (median=725). Margins of non-inferiority were explicitly defined for the non-inferiority studies. All studies employed an intention-to-treat as primary analysis, with one non-inferiority study including per-protocol secondary analysis. On average, superiority studies favoured the novel treatment (OR=0.75), which was not the case for non-inferiority studies (OR=1.31). Few studies swapped between the superiority and non-inferiority approaches as the study progressed, with no evidence that those swaps were in response to the results obtained.

## Conclusion

These active-control studies were found to have appropriate sample size targets, with no evidence of non-inferiority studies being larger. Only one non-inferiority study used per-protocol analysis as suggested in the CONSORT guidelines. There was no evidence of reporting bias due to switches between superiority and non-inferiority approaches.

